# Micromechanical Properties and Deformation Behavior of the Co_70_Fe_3_Mn_3.5_Mo_1.5_Si_11_B_11_ Amorphous Alloy

**DOI:** 10.3390/ma19183946

**Published:** 2026-09-17

**Authors:** Mykhaylo Pashechko, Roman Bilyk, Jaroslaw Borc, Oleksandr Tisov, Nazar Sembratovych, Myroslav Kindrachuk

**Affiliations:** 1 Department of Technical Computer Science, Faculty of Mathematics and Information Technology, Lublin University of Technology, 20-618 Lublin, Poland; 2 Department of Metal Physics, Faculty of Physics, Ivan Franko National University of Lviv, 79005 Lviv, Ukraine; 3 Department of Applied Physics, Faculty of Mechanical Engineering, Lublin University of Technology, 20-618 Lublin, Poland; 4 School of Aerospace Engineering, Xi’an Jiaotong University, Xi’an 710049, China; 5 Department of Applied Mechanics and Materials Engineering, State University “Kyiv Aviation Institute”, 03058 Kyiv, Ukraine

**Keywords:** amorphous metallic glasses, short-range order, micromechanical properties, creep behavior, free volume, nanoindentation

## Abstract

This work reports an experimental study of the micromechanical properties of the Co_70_Fe_3_Mn_3.5_Mo_1.5_Si_11_B_11_ (at. %) amorphous alloy. All experiments were performed at room temperature on the unannealed sample in order to avoid the influence of thermally induced structural relaxation. X-ray diffraction confirmed the amorphous structure. Using nanoindentation tests, we determined key mechanical characteristics under a 20 mN load, including hardness, elastic and plastic deformation parameters, creep ratio, and other micromechanical properties. Scratch testing was performed under both static (20 mN) and dynamic (0.3–100 mN) regimes. We analyzed variations in penetration depth and residual depth during scratch testing. Surface morphology analysis after scratch testing confirmed no macroscopic crack formation or delamination. The friction coefficient behavior indicates sensitivity to localized contact loading and is consistent with localized deformation processes within the sample. A pronounced time-dependent deformation (*C_IT_* ~ 4.7%) under localized contact loading was also observed. The data were analyzed considering the structural state of the sample’s amorphous matrix and existing theories of plastic deformation mechanisms in amorphous systems. The ratio of elastic to plastic deformation, along with time-dependent deformation (creep), enables specification of the mechanical response of the investigated amorphous ribbon under localized microcontact loading. The obtained results provide qualitative insight into the deformation characteristics of unannealed amorphous ribbons.

## 1. Introduction

In materials science, the search for advanced structural materials that combine high strength, wear resistance, thermal stability, and corrosion resistance remains an important task [1,2,3,4,5,6]. Traditional crystalline materials and ceramics often fall short of these requirements because inherent structural defects limit their mechanical performance.

In this context, amorphous alloys, also known as metallic glasses, have attracted considerable attention because of their unique combination of the properties mentioned above [7,8,9,10,11]. These materials possess a disordered atomic structure without long-range order. Their application represents a fundamentally different approach to designing materials with enhanced characteristics. The absence of a crystalline structure eliminates traditional sources of mechanical defects, thereby enabling superior mechanical performance.

The unique combination of physical properties of amorphous metallic alloys makes them promising for applications in precision mechanics, microelectronics, medical implants, and related fields. To date, the structure and key mechanical properties of industrial and model systems, such as Fe- and Zr-based amorphous alloys, have been studied relatively well [12,13,14,15,16,17,18,19,20]. In particular, Zr-based alloys reveal high strength (around 2 GPa) but are brittle and expensive to manufacture [12,13,16,17,18,19]. In contrast, Fe-based alloys offer higher strength (~4 GPa) and economic advantages over the former, but they often exhibit limited plasticity [12,16,17,18]. Thus, the search for systems with targeted properties remains timely.

In this context, Co-based amorphous alloys have attracted particular attention. Literature data indicate that they exhibit higher strength (up to 5–6 GPa) than Fe- and Zr-based amorphous metallic glasses, while also showing greater plasticity than Fe-based systems [12,18,19,20]. However, current publications often focus on studying the influence of individual alloying elements on glass-forming ability and on determining basic magnetic and selected mechanical parameters, such as hardness, Young’s modulus, and the previously mentioned strength, for specific chemical compositions. For example, in [21] it was shown that the Vickers hardness (*HV*) of Co_43-*x*_Cu*_x_*Fe_20_Ta_5.5_B_31.5-*y*_Si*_y_* (*x* = 0, 0.5, 1.0, 1.5; *y* = 5) amorphous alloys changes from 1455 *HV* for Co_43_Fe_20_Ta_5.5_B_31.5_ to 1166 *HV* for Co_41.5_Cu_1.5_Fe_20_Ta_5.5_B_26.5_Si_5_. It was also established that adding Si and Cu to Co–Fe–Ta–B amorphous systems enhances glass-forming ability but simultaneously reduces hardness. For the Co_64.8_Fe_7.2_Mo_4_Si_4.8_B_19.2_ alloy of similar chemical composition and prepared using methods similar to those investigated in the present study, the hardness and Young’s modulus were measured as *H* = 10.81 GPa and *E* = 37.2 GPa, respectively [22].

Nevertheless, systematic data on the micromechanical response under local loading at the microscale in multicomponent amorphous alloys, including Co-based systems, remain a subject of intense investigation.

Understanding such micromechanical behavior also requires appropriate theoretical approaches. The theoretical foundation for understanding the plastic deformation of bulk metallic glasses is based on classical models, such as the shear transformation zone (STZ) model and the free-volume model [23,24]. Although the theoretical models above often partially explain the plasticity of amorphous metallic alloys, their role in the development of creep (i.e., time-dependent deformation) in these materials requires further investigation.

Moreover, currently, relatively few studies specifically examine the creep behavior of amorphous metallic glasses [25]. A quantitative assessment of the underlying mechanisms requires a broader parametric study.

A review of the literature reveals that the key mechanical properties of amorphous alloys depend strongly not only on the content of the base element, chemical composition, and nature of the alloying additions, but also on the features of the atomic structure, which determine the material’s behavior at the micro- and nanoscale. In particular, deformation mechanisms under local contact loading in metallic glasses remain insufficiently studied, underscoring the need for further research. In this paper, we selected the unannealed Co_70_Fe_3_Mn_3.5_Mo_1.5_Si_11_B_11_ amorphous alloy for a detailed investigation of its micromechanical response under localized contact loading, a regime rarely studied in previous work. The high Co content (~70 at. %) is expected to yield higher strength and improved plasticity than Fe-based systems, as suggested by literature data for related Co-based amorphous alloys [12,18,19,20]. Adding Mn and Mo is expected to modify the structural and functional properties of the Co-rich matrix. Mn can influence magnetic characteristics and structural stability, while Mo is associated with enhanced corrosion resistance and structural stability. Although some data exist for similar systems [22], the micromechanical response, particularly creep and deformation under local loading of this alloy, remains unexplored. Additionally, its magnetic softness makes it attractive for microelectronics and sensor applications. The results obtained are analyzed in comparison with other amorphous systems and interpreted in relation to the material’s internal structural features.

## 2. Materials and Methods

The Co_70_Fe_3_Mn_3.5_Mo_1.5_Si_11_B_11_ (at. %) ingot was prepared from high-purity components, including cobalt chips (99.99% purity), iron granules (99.95%), manganese pieces (99.9%), molybdenum flakes (99.9%), crystalline silicon pieces (99.999%), and crystalline boron pieces (99.5%), all supplied by Sigma-Aldrich (St. Louis, MO, USA). Melting was performed in a laboratory vacuum arc furnace (a custom-built water-cooled copper hearth model, built at the Institute of Metal Physics, Kyiv, Ukraine). To ensure both structural and chemical homogeneity, the obtained alloy was remelted four times.

Then, the ingot was subsequently used as the precursor for ribbon fabrication using a custom-built single-roller vacuum melt-spinning system (Institute of Metal Physics, Kyiv, Ukraine). The melt-spinning process was performed under the following operating parameters: the ejection overpressure of argon was maintained at Δ*p* = 25–30 kPa; the nozzle-to-wheel gap was set to 0.5 mm; and the molten alloy was cast onto the clean peripheral surface of a rapidly rotating copper wheel (a custom-built at the Institute of Metal Physics, Kyiv, Ukraine) with a diameter of 300 mm and a linear surface velocity of *v* = 35–40 m/s. This configuration provided a high cooling rate of 10^5^–10^6^ K/s, resulting in continuous amorphous ribbons with a thickness of (20 ± 3) μm.

Its structure was studied using X-ray diffraction. Diffraction patterns were collected over the range 10° ≤ 2θ ≤ 140° using Cu-Kα radiation with a wavelength of approximately 0.154 nm, and the scan rate was 2°/min [26] using a DRON-3 X-ray diffractometer (Research Center “Burevestnik”, modified in Kharkiv, Ukraine). The obtained diffraction patterns were analyzed to confirm the amorphous nature of the investigated sample.

Nanoindentation is a primary research tool for evaluating the mechanical properties and deformation behavior of a wide range of materials at the micro- and nanoscale, including the yield strength of microstructures [27,28,29]. In our study, nanoindentation tests were performed at room temperature using an ultra nanoindentation tester UNHT^3^ (Anton Paar, Graz, Austria) equipped with an optical microscope/camera for test site positioning, operating in linear load mode with the following parameters: test load of 20 mN, loading/unloading rate of 20 mN/min. A holding time of 10 s was used, which allows for determining the creep ratio *C_IT_* with better accuracy [30]. These parameters ensured that the indentation depth was much smaller than the ribbon thickness (<2 μm). A total of 21 indentations were taken for the sample, of which 4 were rejected as drastically deviating from the mean. We used a Berkovich diamond indenter (Anton Paar, Graz, Austria) for microhardness measurements. A built-in optical system was used to capture images of selected indent impressions. The samples were affixed to aluminum discs using adhesive tabs, and the wheel side of the ribbon was investigated.

**Figure 1:**
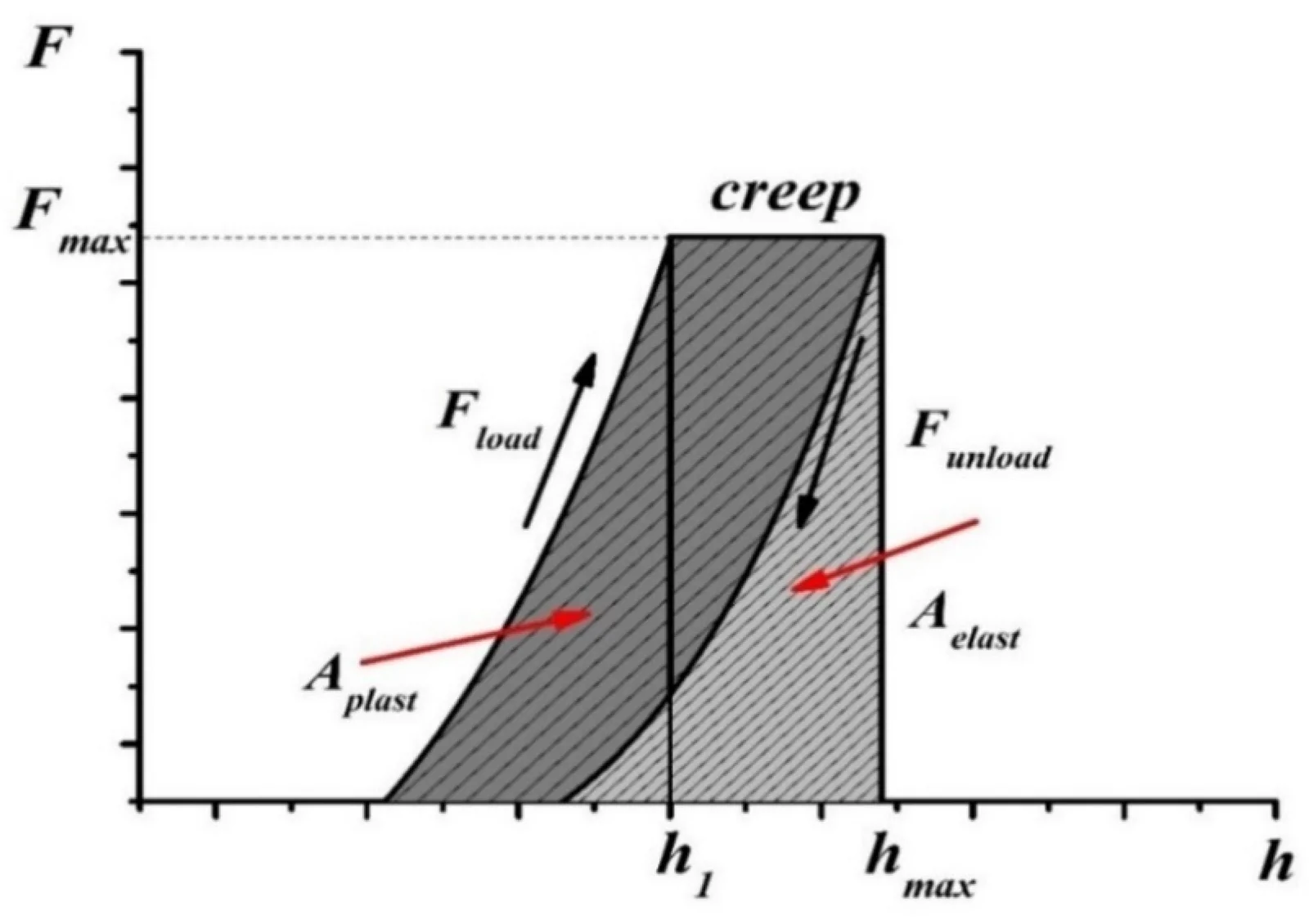
Schematic representation of the *F–h* diagram during nanoindentation.

The mechanical parameters of the sample were calculated from the *F–h* diagrams using the dedicated software package Indentation V9 (Anton Paar, Graz, Austria) included with the UNHT^3^ tester. Figure 1 shows a schematic representation of several measured and calculated values and parameters derived from the *F–h* curves, including the elastic *A_elast_* and plastic *A_plast_* components of mechanical work, creep *C_IT_*, the maximum applied load *F_max_*, the maximum penetration depth *h_max_*, and the indentation depth at the beginning of the holding phase *h*_1_. These parameters, along with others, are discussed in more detail in the Section 3.

Considering possible inhomogeneities at the ribbon surface and in its bulk, a series of measurements was conducted.

The plane strain elastic modulus E* was calculated using the Oliver–Pharr method [31] and was determined according to the following equation:

(1) $$E^{*}=\;\frac{1}{\frac{1}{E_{r}}-\frac{1-\nu_{i}^{2}}{E_{i}}}$$

where *E_i_* (1141 GPa for diamond) and *ν_i_* (0.07 for diamond) are the indenter’s modulus and Poisson’s ratio, *E_r_* is the reduced modulus, which was calculated as

(2) $$E_{r}=\frac{\sqrt{\pi }S}{2\beta \sqrt{A_{p}}}$$

where *β* is the indenter’s geometric correction factor, *A_p_* is the projected contact area of the indentation imprint, and *S* represents the contact stiffness, defined as the derivative at maximum loading.

Another informative parameter is the indentation hardness *H_IT_*, which is determined by the following equation:

(3) $$H_{IT}=\;\frac{F_{max}}{A_{p}}$$

where *F_max_* is the maximum applied load during the test, and *A_p_* is the previously reported contact area.

A more detailed description of the calculation procedure and the determination of other mechanical characteristics can be found in references [32,33].

Scratch tests of the amorphous ribbon surface were carried out using a Nano-Scratch Tester NST^3^ (Anton Paar, Graz, Switzerland) equipped with a friction table and a sphero-conical diamond indenter with a radius of 2 μm [33]. Scratch tests were performed at room temperature in both static and dynamic modes under loads ranging from 0.3 to 100 mN, with a loading rate of 2 mN/s and a horizontal indenter speed of 0.4 mm/min over a scan length of 0.6–0.8 mm. The investigations were performed in 3-scan mode (pre-scan–scratch–post-scan), allowing determination of the scratch depth and the depth after scratching, respectively.

## 3. Results and Discussion

**Figure 2:**
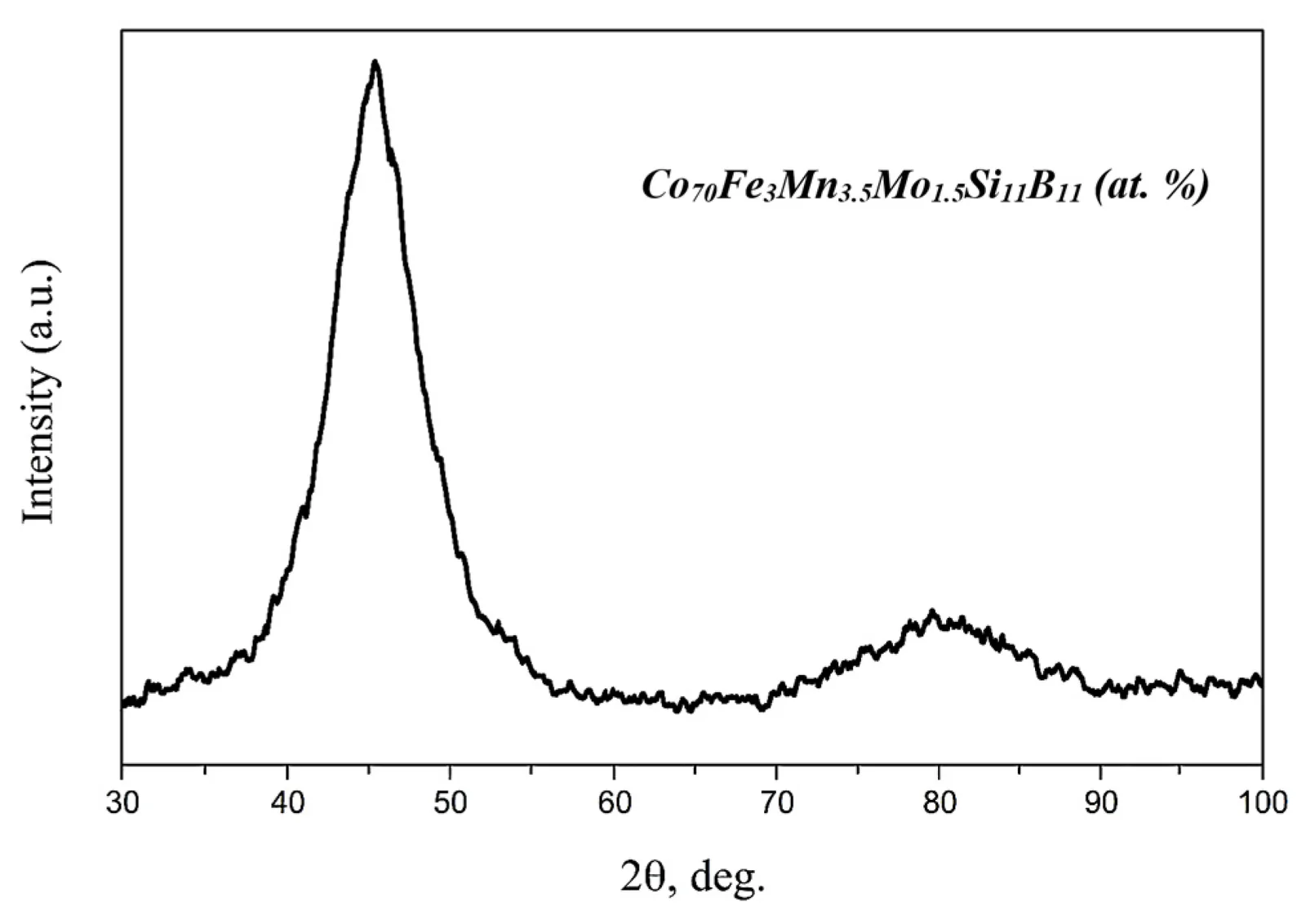
XRD pattern of the investigated Co-based amorphous ribbon.

XRD analysis confirmed the amorphous structure of the Co_70_Fe_3_Mn_3.5_Mo_1.5_Si_11_B_11_ ribbon. The diffraction pattern shows a broad halo peak near 2θ ≈ 45°, accompanied by a weaker, broader hump near 2θ ≈ 80°, with no sharp peaks typical of crystalline phases (Figure 2). This diffraction pattern is typical of amorphous materials. The position and width of these diffuse maxima reflect the short-range order in the investigated ribbon.

To determine the mechanical properties of Co_70_Fe_3_Mn_3.5_Mo_1.5_Si_11_B_11_, tribological studies were performed, including nanoindentation and nanoscratch experiments, following the methodologies and parameters described in the previous section. All measurements were performed in the unannealed state, excluding the influence of structural relaxation or nanocrystallization. Thermal treatment could induce such phenomena and significantly change the mechanical response [34,35,36].

**Figure 3:**
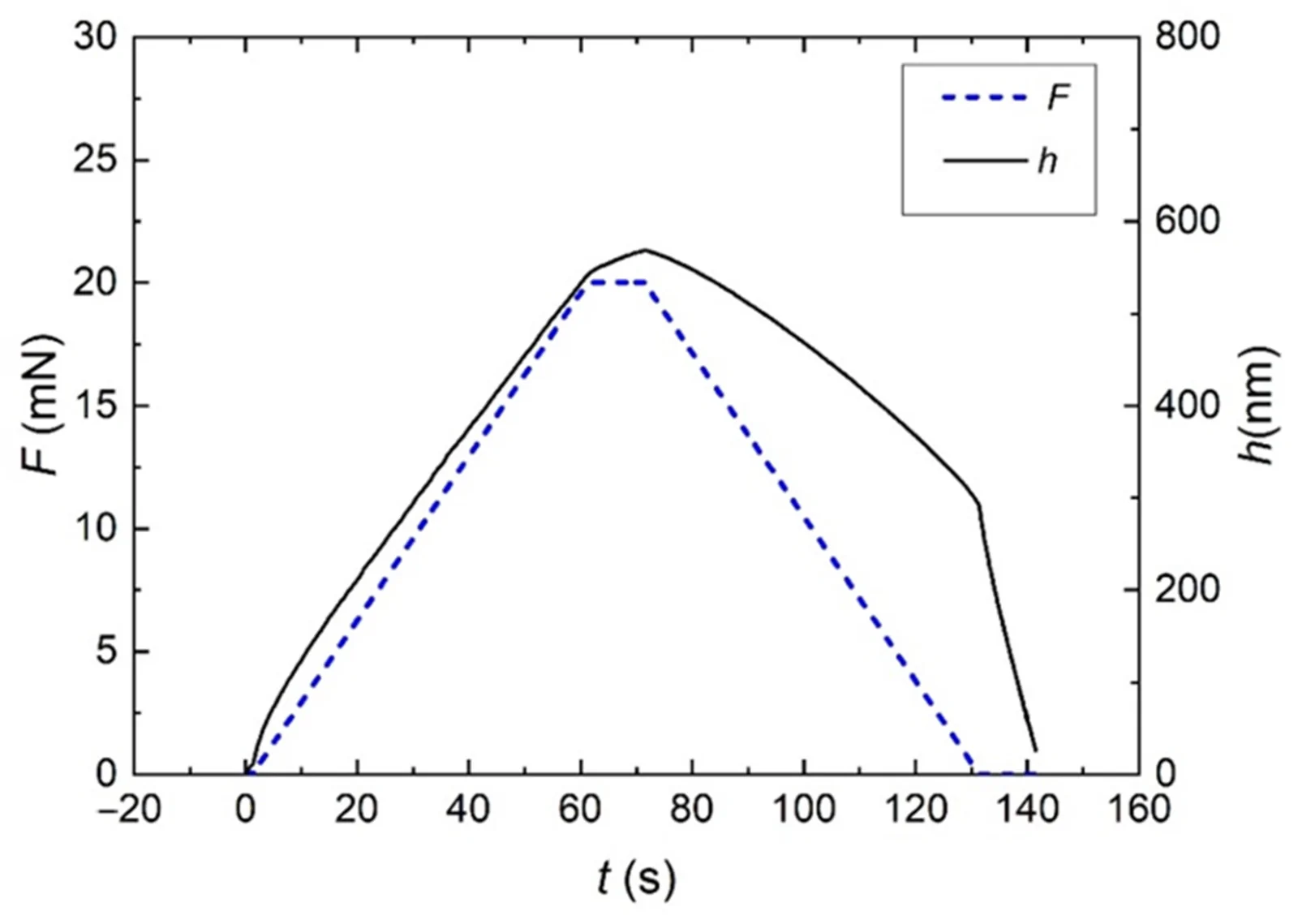
Load *F*(*t*) and penetration depth *h*(*t*) curves for the Co_70_Fe_3_Mn_3.5_Mo_1.5_Si_11_B_11_ amorphous ribbon.

**Figure 4:**
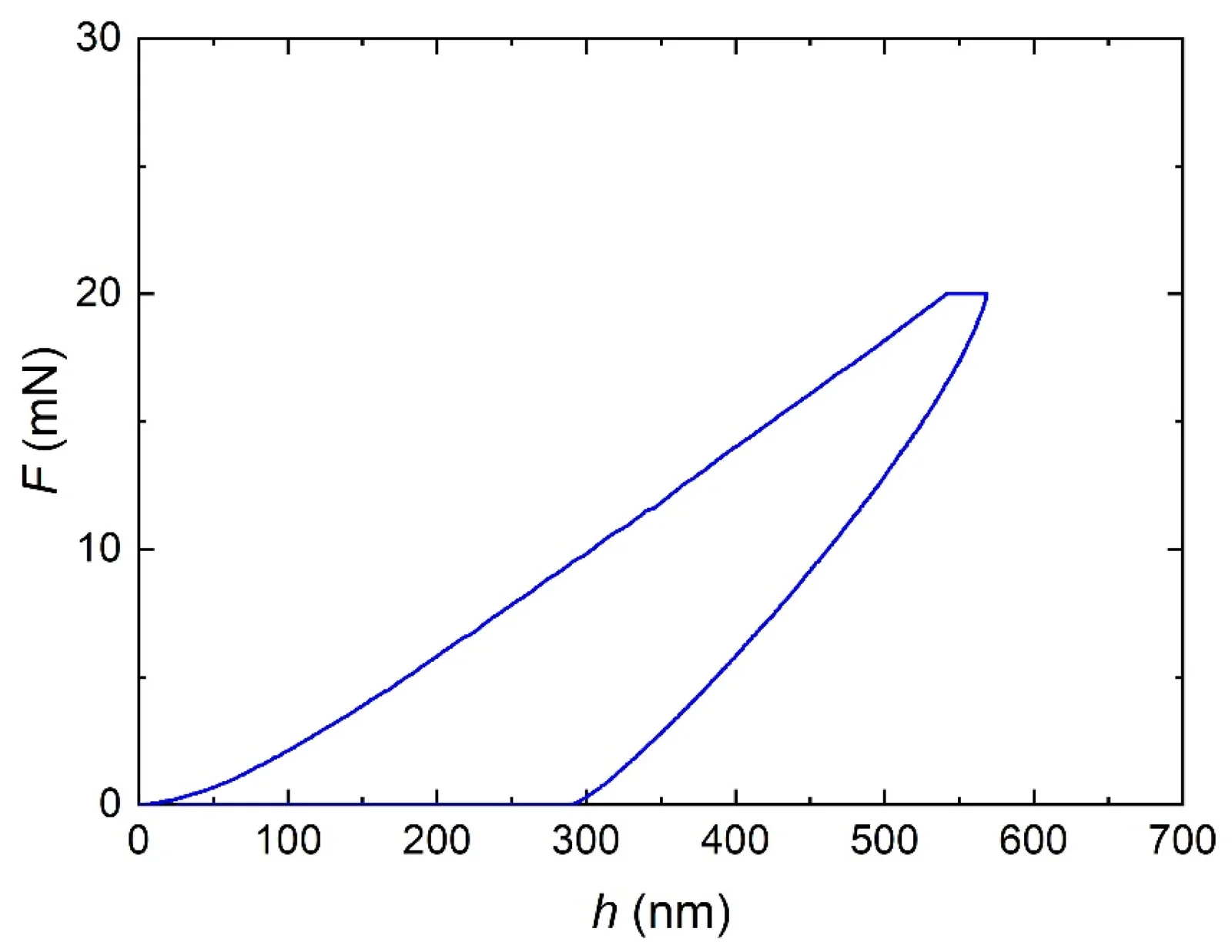
A representative loading–unloading kinetic curve *F-h* for the amorphous Co_70_Fe_3_Mn_3.5_Mo_1.5_Si_11_B_11_ ribbon.

From the obtained load–unload indentation curves (Figure 3 and Figure 4), a set of micromechanical parameters was determined. Table 1 presents some averaged values. In particular, the average hardness *H_IT_* of the Co_70_Fe_3_Mn_3.5_Mo_1.5_Si_11_B_11_ alloy was (4.817 ± 0.616) GPa. The average plane-strain modulus was (49.46 ± 5.85) GPa, indicating a significant contact microscale elastic response, although lower than that of crystalline alloys. The obtained values of *H* and *E* are comparable to those reported for similar Co–Fe–Mo–Si–B amorphous alloys [22]. Furthermore, our sample is structurally unrelaxed. This results in an increased average interatomic spacing, thereby decreasing the elastic modulus relative to structurally relaxed ingots.

In addition, the mechanical response can be described using the *H/E* and *H*^3^*/E*^2^ ratios, which serve as qualitative indicators of the material’s resistance to deformation and crack propagation under contact loading [37]. These parameters are empirical. The investigated Co-based ribbon exhibits relatively high *H/E* (~0.1). This ratio in our sample is partly due to its structurally unrelaxed state, which leads to expanded average interatomic spacing. This combination of hardness and elastic modulus indicates a material that can better accommodate localized contact stresses through elastic deformation.

The *H*^3^*/E*^2^ parameter is associated with resistance to plastic deformation and correlates with crack resistance. In the present case, the obtained value of *H*^3^*/E*^2^ (~0.05 GPa) correlates with the absence of cracking observed during scratch testing, as discussed in detail below.

Another important parameter in nanoindentation tests is the contact stiffness *S*, which represents the material’s ability to resist local deformation at the indenter-sample interface. It depends, among other factors, on the sample’s deformation behavior and the indenter geometry. A higher *S* value indicates a stiffer sample at the contact point and lower deformation. In this study, the average contact stiffness was (0.113 ± 0.009) mN/nm, with minimal variation across the tested contact area, suggesting a relatively uniform mechanical response.

The indentation data for the amorphous Co_70_Fe_3_Mn_3.5_Mo_1.5_Si_11_B_11_ system enabled the determination of the elastic and plastic components of the indentation work, which were subsequently averaged. In these load–depth curves (an experimental example shown in Figure 4 and schematically explained in Figure 1), the upper part of the curve characterizes the resistance to indentation and is associated with plastic deformation, while the lower part reflects the elastic recovery of the sample. The area under these curves represents the plastic and elastic indentation work, respectively.

The analysis of deformation work showed that the total nanoindentation work, *A_total_,* was 5532.7 pJ, of which the plastic component, *A_plast_* = 3140.8 pJ, indicates a substantial contribution of plastic deformation, and the elastic component, *A_elast_* = 2391.9 pJ, confirms partial elastic recovery upon unloading. The relaxation ratio *p* (≈43%) characterizes the material’s relaxation ability and indicates that a significant part of the energy is spent on plastic deformation (~57%), which agrees with other measured data and reflects the material’s moderate elastic response. This can be useful when some energy needs to be absorbed in the microcontact zone.

As shown in Figure 4, a pronounced time-dependent increase in penetration depth is observed during the load-hold segment of the indentation test, indicating a measurable indentation creep response. This behavior is quantified by the indentation creep ratio *C_IT_*, which is defined according to [38] as *C_IT_* = (*h_max_* − *h*_1_*)*/*h*_1_ (see Figure 1 and Figure 4).

Compared to common crystalline metals such as stainless steel, Al-alloys, and Cu–Cr–Zr, which typically exhibit low indentation creep values under room-temperature conditions (*C_IT_* ≈ 0.5–1.2%) [39], the relatively elevated *C_IT_* (~4.7%) obtained in the present work indicates an enhanced susceptibility to time-dependent deformation under localized contact loading. Similar behavior has been reported for Co(Fe)-based metallic glasses, where time-dependent deformation under nanoindentation is commonly associated with stress-assisted atomic rearrangements activated within the amorphous structure [40]. In the present work, the alloy is studied without annealing, meaning that its atomic structure has not fully stabilized and contains some structural inhomogeneity. When local stress is applied by the indenter, this inhomogeneity can cause atoms to rearrange more easily, resulting in a continuous increase in penetration depth during the holding period. It is important to note that, like the contact stiffness *S*, the *C_IT_* is a contact parameter. As was reported in [26,40,41,42], creep behavior in some amorphous systems is highly influenced by the applied loading rate during indentation, although a detailed investigation of this effect lies beyond the scope of the present study. However, while this interpretation is consistent with the existing literature, a more detailed parametric analysis is required to quantitatively describe the underlying mechanisms of creep behavior.

Nevertheless, the observed *C_IT_* value provides clear experimental evidence of a pronounced time-dependent deformation in the micromechanical response of the investigated Co-based amorphous ribbon under localized contact loading.

**Figure 5:**
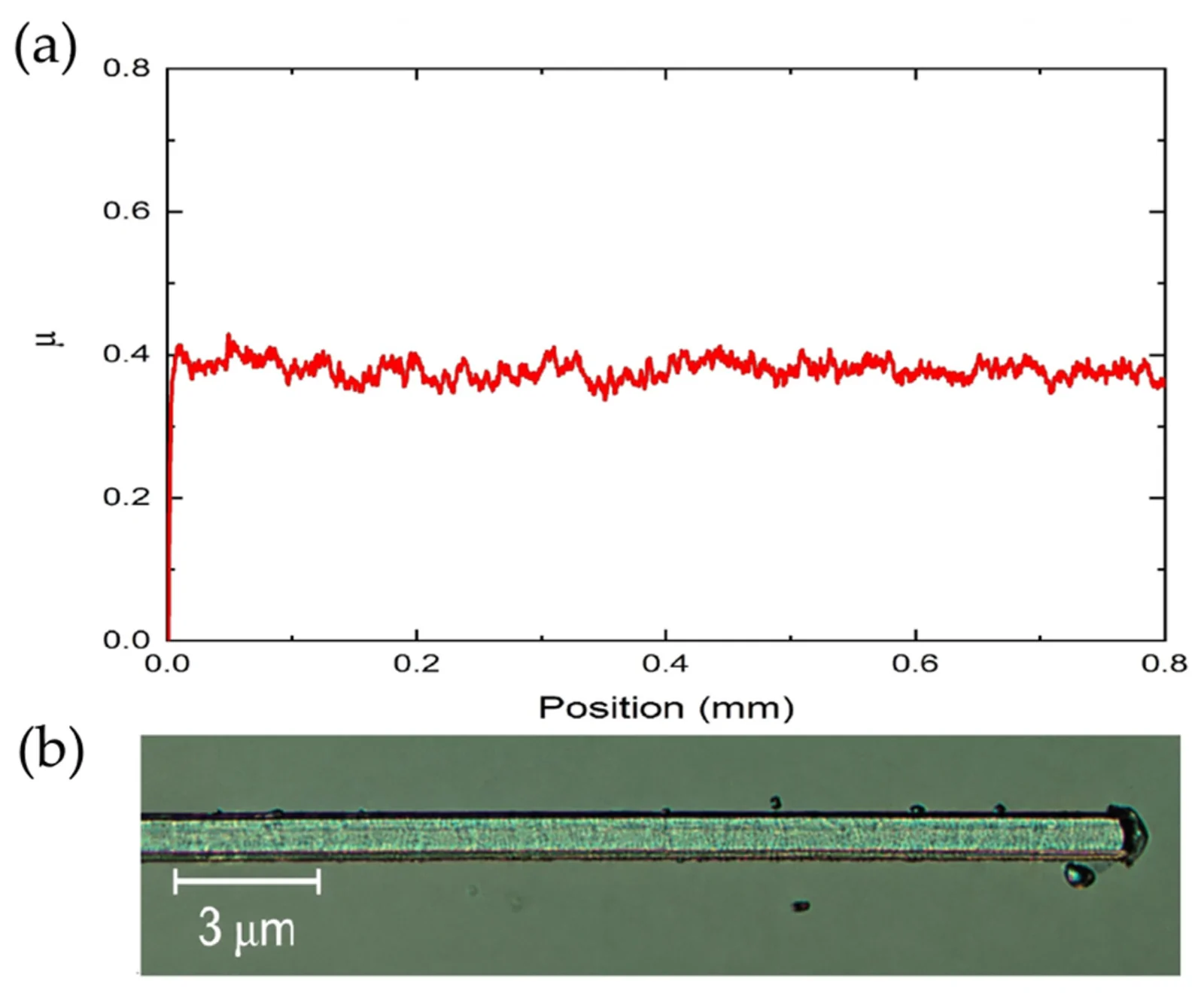
(**a**) The friction coefficient profile and (**b**) micrograph of the scratch track for the amorphous Co_70_Fe_3_Mn_3.5_Mo_1.5_Si_11_B_11_ alloy at 20 mN load.

To further probe the mechanical response of the surface layer under the indenter, scratch testing was performed under both static and dynamic loading regimes. Figure 5 presents a micrograph of the surface morphology (with a 3 μm scale bar) of the investigated amorphous ribbon along with the corresponding friction coefficient curve obtained during the scratch tests conducted at a constant normal load of 20 mN. The indenter trace showed no discontinuities. The absence of defects or cracks along the scratch track suggests a homogeneous surface structure of the amorphous system and indicates a stable mechanical response of the surface layer under the indenter.

**Figure 6:**
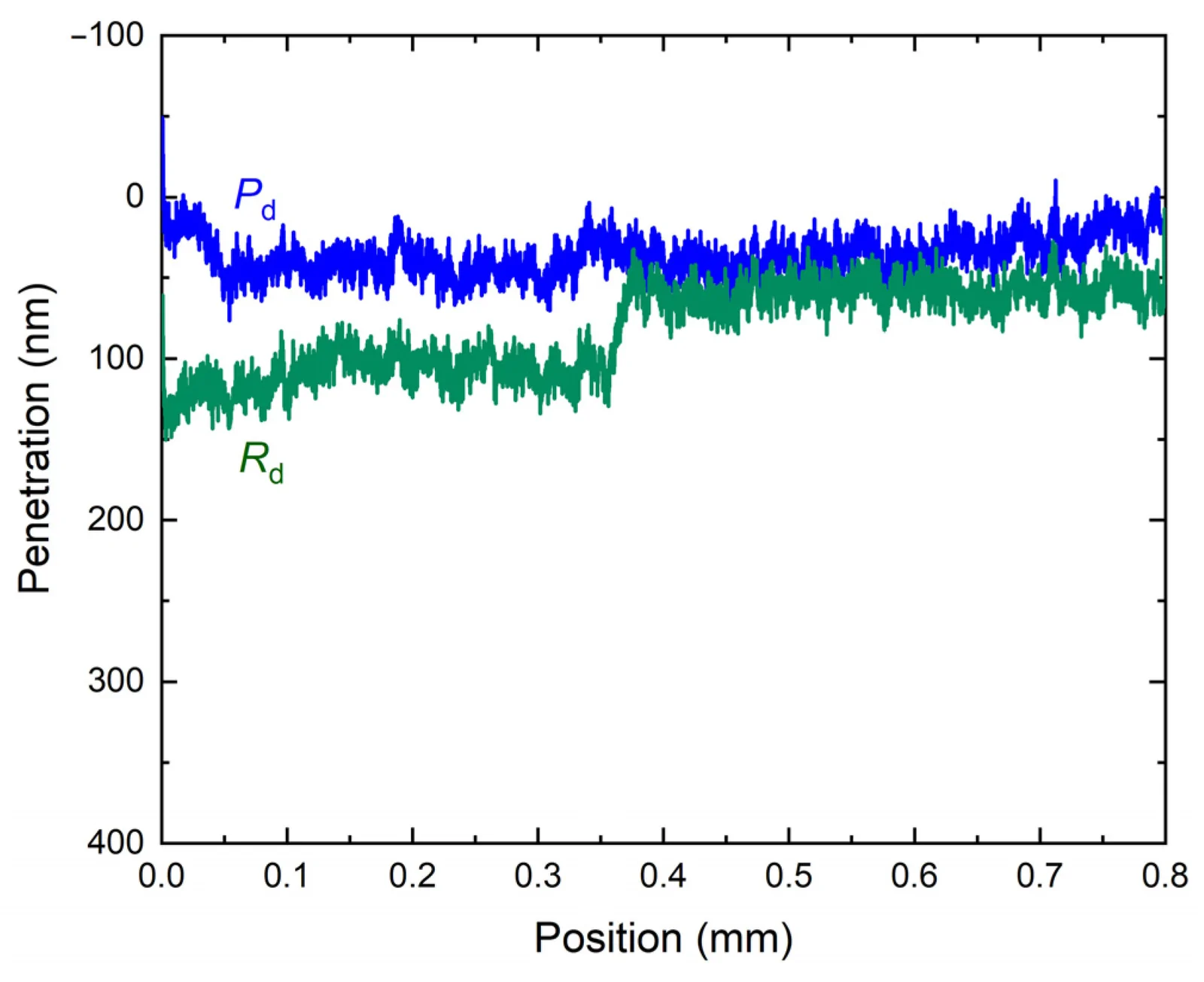
Penetration *Pd* and residual *Rd* depth profiles in scratch testing of the amorphous Co_70_Fe_3_Mn_3.5_Mo_1.5_Si_11_B_11_ alloy at 20 mN load.

This is consistent with the smooth profile of the friction coefficient (Figure 5a). The friction coefficient as a function of normal load shows oscillatory behavior, with no pronounced peaks, indicating the absence of mechanical failure in the sample [43]. Furthermore, the friction coefficient reaches a quasi-steady-state plateau, indicating a stable slip regime. This observation is consistent with the surface morphology of the investigated amorphous alloy and the behavior of the penetration depth (*Pd*) and residual depth (*Rd*) profiles measured at a normal load of 20 mN (Figure 6).

As shown in Figure 6, both curves remain stable throughout the scratch test, as evidenced by the absence of sharp jumps or peaks. This also confirms the material’s local structural stability.

**Figure 7:**
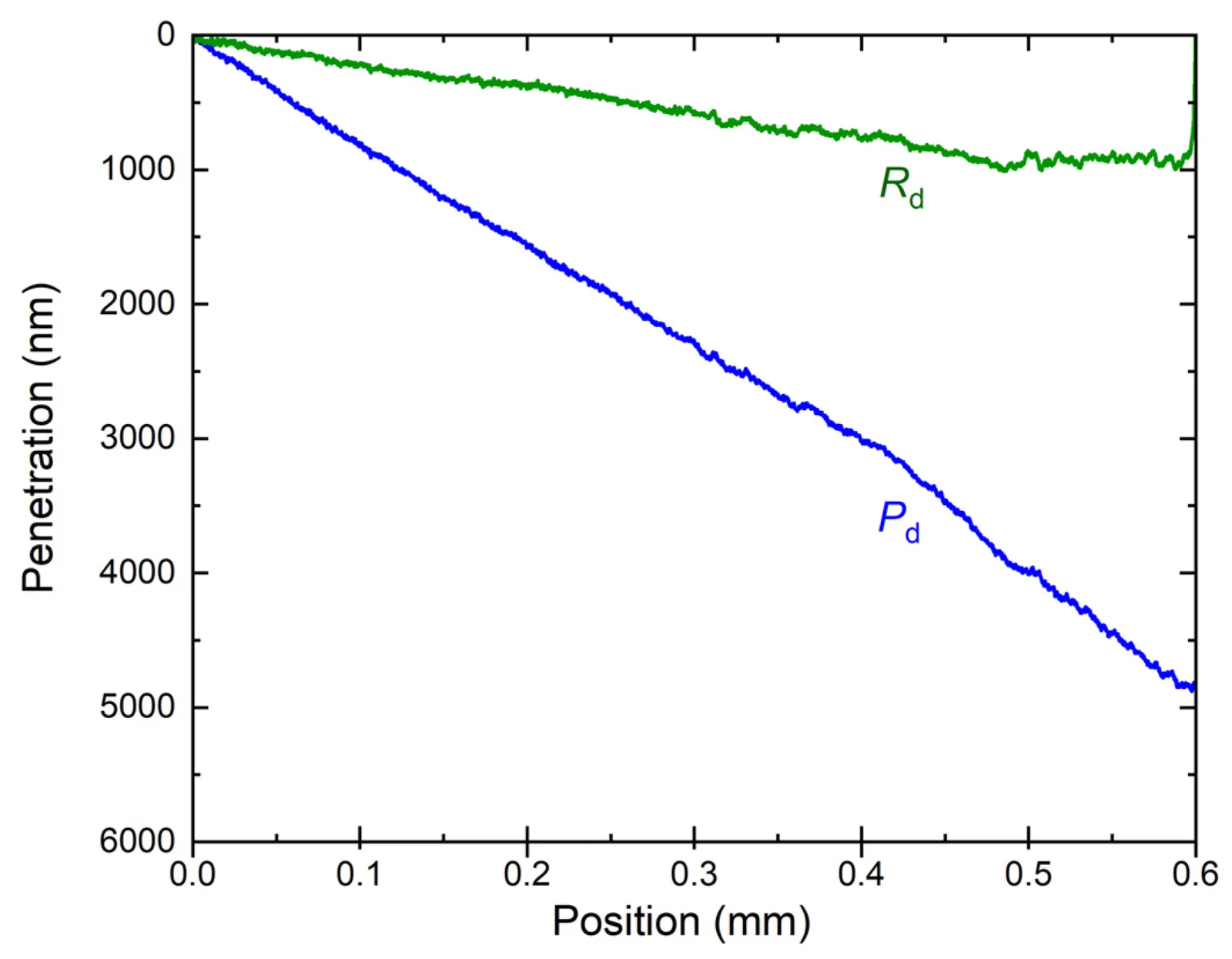
Penetration depth *Pd* and residual depth *Rd* in scratch testing of the amorphous Co_70_Fe_3_Mn_3.5_Mo_1.5_Si_11_B_11_ ribbon under dynamic loading.

Under dynamic loading ranging from 0.3 mN to 100 mN, a similar behavior of *Pd* and *Rd* is observed (Figure 7). As seen, both *Pd* and *Rd* increase nearly linearly with increasing load, though at different rates, and without any discontinuities, similarly to the static loading mode.

This behavior indicates continuous indenter penetration without sudden displacement, confirms the local absence of brittle fracture, and demonstrates the mechanical stability of the sample under the applied dynamic loading regime. At the maximum load of 100 mN, the penetration depth *Pd* reaches approximately 5000 nm, while the residual depth *Rd* is about 750 nm. The smooth character of the profiles is also consistent with the absence of macroscopic signs of brittle fracture during scratching. However, the detailed deformation mechanisms require further nanoscale analysis.

The local absence of brittle fracture and the observed dispersion of plastic deformation can be explained using the STZ concept. The structurally unstable, unannealed state is likely associated with a higher concentration and a more homogeneous distribution of free volume than the annealed state. This reduces the critical stress required for STZ activation. Under the indenter, this promotes the simultaneous activation of numerous small STZs throughout the volume, rather than the localization of deformation into a single macroscopic shear band. Such dispersion of plastic flow inhibits the formation of a critical crack and macroscopic failure, as observed experimentally. This picture is further supported by the material’s micromechanical indices. The obtained value of *C_IT_* ≈ 4.7% is also consistent with this picture, indicating the facile activation of time-dependent creep in such a structure.

These observations collectively indicate that the surface layer of the unannealed ribbon can accommodate localized contact stresses without mechanical damage, whether under static or dynamic load. This behavior, due to the material’s non-equilibrium structure, explains the stable mechanical response observed across different testing modes. This conclusion is also supported by the behavior of the friction coefficient under dynamic loading, which is discussed below.

**Figure 8:**
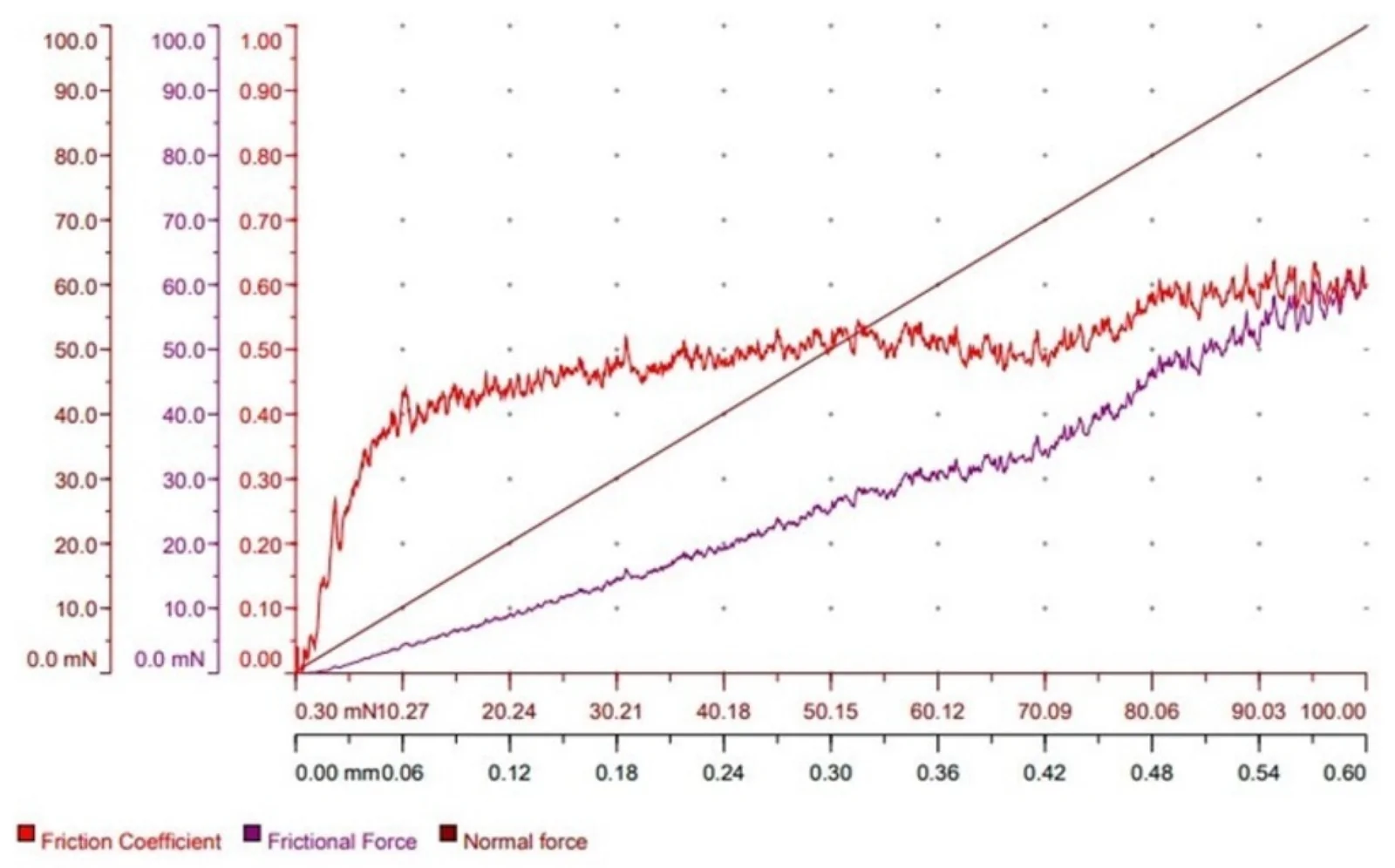
Friction force *F_f_* and friction coefficient *μ* versus applied normal load during dynamic scratch testing of the Co_70_Fe_3_Mn_3.5_Mo_1.5_Si_11_B_11_ amorphous ribbon.

The final stage of this study involved evaluating the local frictional properties of the amorphous Co_70_Fe_3_Mn_3.5_Mo_1.5_Si_11_B_11_ alloy using scratch testing under dynamic loading. The results are shown in Figure 8.

As shown in Figure 8, the applied normal load graph shows no irregularities and fully corresponds to the programmed linear loading mode. During the scratch test, the friction force increases approximately linearly. However, some fluctuations are seen as the scratch length and normal load increase. Similar behavior, known as the stick-slip phenomenon of the friction force curve under applied load along the scratch trace, is observed in other amorphous systems as well [44,45].

The behavior of the friction coefficient also reveals the sensitivity of the sample’s surface to local loading. At the initial stage (up to 0.06 mm), an increase in this parameter is observed, which is attributed to the establishment of stable contact between the indenter and the surface of the investigated ribbon. Saturation of the friction coefficient is observed after 0.1 mm and at a load of around 20 mN, corresponding to the stable sliding stage. Each fluctuation in the friction force corresponds to a synchronous change in the friction coefficient, ultimately forming a so-called saw-tooth profile. This, as in the case of the friction force, is explained by the formation of shear bands in the investigated amorphous material.

## 4. Conclusions

This paper studied the micromechanical response of the unannealed Co_70_Fe_3_Mn_3.5_Mo_1.5_Si_11_B_11_ amorphous ribbon. The non-equilibrium atomic structure characteristic of this state facilitates an investigation of deformation behavior under applied contact loading.

Nanoindentation results show that the investigated alloy exhibits an average hardness HIT (~4.8 GPa) and a modulus E* (~49.5 GPa), comparable to those of many structurally relaxed Co-based amorphous systems. Scratch testing confirmed a stable response of the ribbon under both static and dynamic loading. Under static load, smooth profiles of penetration *Pd* and residual *Rd* depth, along with a stable friction coefficient, indicated the local absence of brittle fracture or delamination. In both regimes, the friction coefficient shows stable evolution with minor fluctuations, indicating localized deformation without a macroscopic crack. When the applied force does not exceed 20 mN, the penetration depth does not exceed 8% of the sample thickness. For forces exceeding 30 mN, the substrate effect could be taken into account. However, this effect has not been investigated.

This is also consistent with other nanoindentation data, in particular the larger contribution of plastic deformation (~57%) compared to elastic deformation (~43%), as well as the measured creep value *C_IT_* (~4.7%), which indicates increased activation of atomic rearrangements under load. This mechanism is responsible for the distributed plastic flow, preventing brittle failure.

Overall, these results provide experimental insight into the micromechanical behavior of the unannealed Co_70_Fe_3_Mn_3.5_Mo_1.5_Si_11_B_11_ amorphous ribbon. They confirm the importance of structural non-equilibrium in forming the micromechanical response to localized mechanical contact and extend experimental knowledge of amorphous systems under localized loading. The findings may be relevant for applications involving microscale mechanical contact of Co-based amorphous ribbons of the investigated composition without prior thermal relaxation. A more detailed parametric study would be required to quantitatively identify the underlying mechanisms.

## Figures and Tables

**Table 1:** Some average micromechanical characteristics of the Co_70_Fe_3_Mn_3.5_Mo_1.5_Si_11_B_11_ alloy.

**Indentation hardness** ***H_IT_*** **, GPa**	**Vickers microhardness *HV*_0.002_**	**Plane strain modulus** E*, GPa	**Reduced modulus** ***E_r_*** **, GPa**	
1	2	3	4	
4.817 ± 0.616	446.1 ± 57.1	49.46 ± 5.85	47.39 ± 5.39	
**Elastic work** ***A_el_*** **, pJ**	**Plastic work** ***A_pl_*** **, pJ**	**Total work** ***A_total_*** **, pJ**	**Relaxation ability** ***p*** **= *A_el_*/*A_total_*, %**	**Plasticity coefficient** ***R*** **= *A_pl_*/*A_total_*, %**
5	6	7	8	9
2391.9 ± 231.7	3140.8 ± 251.4	5532.7 ± 419.6	43.23	56.80
**Maximal indentation depth *h*_1_, nm**	**Maximal load** ***F*** **_max_** **, mN**	**Contact stiffness** ***S*** **, mN/nm**	**Creep’s ratio *C_IT_*, %**	
10	11	12	13	
588.7 ± 34.2	20.00	0.113 ± 0.009	4.73 ± 0.43	

## Data Availability

The original contributions presented in this study are included in the article. Further inquiries can be directed to the corresponding authors.
